# Implementation of Health Technology Assessment in the Middle East and North Africa: Comparison Between the Current and Preferred Status

**DOI:** 10.3389/fphar.2020.00015

**Published:** 2020-02-21

**Authors:** Ahmad Fasseeh, Rita Karam, Mouna Jameleddine, Mohsen George, Finn Børlum Kristensen, Abeer A. Al-Rabayah, Abdulaziz H. Alsaggabi, Maha El Rabbat, Maryam S. Alowayesh, Julia Chamova, Adham Ismail, Sherif Abaza, Zoltán Kaló

**Affiliations:** ^1^Doctoral School of Sociology, Faculty of Social Sciences, Eötvös Loránd University Budapest, Budapest, Hungary; ^2^Syreon Middle East, Alexandria, Egypt; ^3^Faculty of Sciences and Medical Sciences, Lebanese University, Hadath, Lebanon; ^4^Health Technology Assessment Department, National Authority for Assessment & Accreditation in Healthcare (INEAS), Tunis, Tunisia; ^5^Universal Health Insurance Authority, Cairo, Egypt; ^6^Faculty of Health Sciences, Department of Public Health, University of Southern Denmark, Odense, Denmark; ^7^Department of Pharmacy, Center for Drug Policy & Technology Assessment (CDPTA), King Hussein Cancer Center, Amman, Jordan; ^8^King Saud Bin Abdulaziz University for Health Sciences, Ministry of National Guard Health Affairs, Riyad, Saudi Arabia; ^9^Faculty of Medicine, Cairo University, Cairo, Egypt; ^10^Middle East and North Africa Health Policy Forum, Cairo, Egypt; ^11^Department of Pharmacy Practice, Faculty of Pharmacy, Kuwait University, Jabriyah, Kuwait; ^12^Global Networks, ISPOR, Lawrenceville, NJ, USA; ^13^Regional Office for the Eastern Mediterranean, World Health Organization, Cairo, Egypt; ^14^Center for Health Technology Assessment, Semmelweis University, Budapest, Hungary; ^15^Syreon Research Institute, Budapest, Hungary

**Keywords:** health technology assessment, economic evaluation, evidence-based health policy, Middle East and North Africa, HTA implementation

## Abstract

**Introduction:**

Implementation of health technology assessment (HTA) is still in an early stage with some heterogeneity in the Middle East and North Africa (MENA). Our objective was to assess the current and future status of HTA implementation in the MENA region by focusing on regional commonalities.

**Methods:**

Preparatory discussions for the first ISPOR conference in the MENA region indicated some potentially generalizable trends of HTA roadmaps. To widen the perspective, a policy survey was conducted among conference participants by applying an HTA implementation scorecard. Discussion group members helped to validate key conclusions during and after the conference.

**Results:**

Health policy experts in MENA countries would like to facilitate HTA implementation and expect significant changes with some generalizable directions in 10 years compared to the current status according. HTA capacity building has to be strengthened by more graduate and postgraduate programs. Increased public budget and enhanced institutionalization are necessary success factors of HTA implementation. The scope of HTA has to be extended from pharmaceuticals to non-pharmaceutical technologies and to revision of previous policy decisions. Although cost-effectiveness with explicit threshold remains the most preferred HTA criterion, several other criteria have to be considered, maybe even by applying an explicit MCDA framework. The role of local evidence and data has to be strengthened in MENA countries, which translates to the extended use of local patient registries and payers' databases. Duplication of efforts can be reduced if international collaboration is integrated into national HTA implementation.

**Discussion:**

Our results should be viewed as an initial step in a multi-stakeholder dialogue on HTA implementation. Each MENA country should develop its context-specific HTA roadmap, as such roadmaps are not transferable without taking into account country size, economic status, public health priorities and adopted systems of health care financing.

## Introduction

The economic status of countries in the Middle East and North Africa (MENA) are heterogenous, as the region includes some of the highest income countries globally alongside several low- and middle-income countries (Yorulmaz., 2016). Nonetheless, several commonalities are shared culturally and specifically in health care systems, including fragmentation of health care provision and financing and efforts to implement universal health coverage. Budget constraints are on the health policy agenda in almost all countries due to several reasons among which; political instability, refugee crisis upscaled economic problems of several low- and middle-income countries. In the recent era of lower oil prices, even high-income Gulf countries are also forced to rethink their public policies, including health policies toward a more cost-conscious direction (Pharmaceuticals and Healthcare Outlook for 2017: Middle East & North Africa, 2016).

Whether the objective is to rationalize health care expenditure or increase return on investment, the term Health Technology Assessment (HTA) started to gain attention in countries of the region. HTA refers to the systematic evaluation of the properties and effects of health technology, addressing the direct and intended effects of this technology, as well as its indirect and unintended consequences, and aimed mainly at informing decision making regarding health technologies. HTA is conducted by interdisciplinary groups that use explicit analytical frameworks drawing on a variety of methods (International Network of Agencies for Health Technology Assessment, 2006). The main purpose of conducting HTA is to inform health care policy-makers and improve the evidence base of policy decisions. Although HTA has been increasingly considered to support health policy decisions in the MENA region, some of the core HTA components, specifically economic evidence is not heavily and formally utilized in pricing and reimbursement decisions of health technologies (World Health Organization, 2015). While HTA implementation strategies and experiences from other countries can provide guidance, HTA roadmaps are not still fully transferable (Kaló et al., 2016). No two HTA systems are the same and no general variables (including demographics, health status, geography, GDP, fragmentation of health care financing) explain major variations in HTA implementation (Löblová et al., 2019). Our objective was to provide an overview on the current status of HTA implementation in the Middle East and North Africa (MENA) region and to identify and recommend objectives for the next 10 years by focusing on regional commonalities, and not on differences across countries.

## Methodology

The International Society of Health Economics and Outcomes Research (ISPOR) organized its first MENA regional conference in Dubai in September 2018. Several satellite meetings were adjusted to the ISPOR Dubai 2018 conference, including the 2^nd^ Middle East and North Africa Health Policy Forum.

HTA implementation in the MENA region was one of the main topics of the ISPOR Dubai 2018 conference and the satellite MENA Health Policy Forum. The preparatory work for these events included several meetings and teleconferences among HTA experts representing different ISPOR teams and MENA organizations. These discussions were so rich in potentially generalizable conclusions, which resulted in a consensus among experts to summarize the current and future status of HTA implementation in the MENA region in a health policy paper.

In order to widen the perspective, a policy survey was conducted by applying an HTA implementation scorecard that had been designed to support the formulation of HTA roadmaps in developing countries (Kaló et al., 2016). Current status and future preferences for HTA implementation were explored in eight areas: capacity building; HTA financing; process and organizational structure; scope of HTA; decision criteria, standardization of methodology; use of local data; and international collaboration.

The survey was distributed in an electronic format to registrants of the ISPOR Dubai conference a few days before the event in September 2018. To expand the respondents base, leaders of ISPOR chapters in the MENA region were encouraged to share the link of the survey with other HTA experts not registered in the conference. A paper-based version of the same survey was distributed during the onsite registration for those conference participants, who had not previously filled in the online survey.

The survey was anonymous, and participants consented that their individual survey responses can be aggregated and used in scientific presentations and publications. The paper-based version of the survey is listed in **Appendix 1**. Survey response was considered valid if not more than four answers were missing or invalid (e.g., providing multiple answers for a single-choice question, or not answering a question). Also, survey responses from outside the MENA region were excluded. Results of invalid surveys or responses were excluded from the aggregated data set.

Preliminary survey results were presented during the ISPOR Dubai 2018 conference in a policy panel with senior HTA experts from Egypt, Jordan, Kuwait, Lebanon, and Tunisia. The discussion was led by two international HTA professionals from outside the region. Comments of panelists were transcribed and incorporated in the paper.

After the conference, coauthors helped to validate key conclusions of the manuscript by providing published references and anecdotal evidences in writing.

## Results

Participants from 11 countries contributed with valid responses to the survey (see **Table 1**). More than two-third of respondents were from Saudi Arabia, Egypt, Jordan, and Lebanon. Less than four respondents represented Kuwait, United Arab Emirates, Tunisia, Oman, Iran, Yemen, and Qatar.

**Table 1:** Demographics of survey respondents.

Main employment	
Public sector	28 (54.9%)
Private sector	23 (45.1%)
**Major training**	
Economics	4 (7.8%)
Pharmacy	25 (49.0%)
Medicine	8 (15.7%)
Other health care (e.g., nursing, dietetics)	6 (11.8%)
Multidisciplinary (at least two master's degrees from the above list)	5 (9.8%)
Other	3 (5.9%)
**Age**	
Below 30	9 (17.6%)
Between 30 and 50	32 (62.7%)
Above 50	10 (19.6%)

After exclusion of 39 non-valid responses, 51 responses were aggregated. 55% of the respondents were from the public and academic sector compared to 45% from the private sector, including pharmaceutical or medical device manufacturers. About half of the respondents (49%) had primary education in pharmaceutical sciences. Detailed results of the survey respondents are presented in **Table 2**.

**Table 2:** Aggregated results of valid responses from HTA implementation survey.

	Current	Preferred
**1. HTA Capacity Building**		
**a) Education**		
No training	14 (29.2%)	2 (4.0%)
Project based training and short courses	18 (35.4%)	2 (4.0%)
Permanent graduate program with short courses	5 (10.4%)	8 (16.0%)
Permanent graduate and postgraduate program with short courses	13 (25.0%)	39 (76.0%)
**2. HTA Funding**		
**a) Financing critical appraisal of technology assessment**		
No funding for critical appraisal of technology assessment reports or submissions	41 (78.0%)	4 (7.8%)
Dominantly private funding (e.g., submission fees) by manufacturers for the critical appraisal of technology assessment reports or submissions	8 (14.0%)	12 (21.6%)
Dominantly public funding for critical appraisal of technology assessment reports or submissions	4 (8.0%)	37 (70.6%)
**b) Financing health technology assessment (i.e., HTA research)**		
No public funding for technology assessment; private funding is not needed or expected	27 (52.0%)	5 (9.8%)
No or marginal public funding for research in HTA; private funding is expected	19 (38.0%)	7 (11.8%)
Sufficient public funding for research in HTA; private funding is also expected	2 (4.0%)	20 (39.2%)
HTA research is dominantly funded from public resources	5 (6.0%)	21 (39.2%)
**3. Legislation on HTA**		
**a) Legislation on the role of HTA process and recommendations in decision-making process**		
No formal role of HTA in decision-making	27 (55.3%)	4 (8.7%)
Dominantly international HTA evidence is taken into account in decision-making	17 (36.2%)	2 (4.3%)
International and additionally local HTA evidence is taken into account in decision-making	4 (8.5%)	22 (47.8%)
Local HTA evidence is mandatory in decision-making	1 (0.0%)	19 (39.1%)
**b) Legislation on organizational structure for HTA appraisal**		
There is no public committee or institute for the appraisal process	31 (58.8%)	5 (9.8%)
Committee is appointed for the appraisal process	12 (21.6%)	2 (3.9%)
Committee is appointed for the appraisal process with support of academic centers and independent expert groups	2 (2.0%)	3 (5.9%)
A public HTA institute or agency is established to conduct formal appraisal of HTA reports or submissions	2 (3.9%)	3 (5.9%)
Public HTA institute or agency is established to conduct formal appraisal of HTA reports or submissions with support of academic centers and independent expert groups	3 (5.9%)	22 (43.1%)
Several public HTA bodies are established without central coordination of their activities	4 (7.8%)	1 (2.0%)
Several public HTA bodies are established with central coordination of their activities	0 (0.0%)	17 (29.4%)
**4. Scope of HTA Implementation**		
**a) Scope of technologies (multiple choice)**		
HTA is not applied to any health technologies	26 (51.0%)	4 (4.0%)
Pharmaceutical products	24 (49.0%)	37 (92.0%)
Medical devices	7 (14.3%)	37 (78.0%)
Prevention programs and technologies	2 (4.1%)	34 (66.0%)
Surgical interventions	1 (2.0%)	34 (64.0%)
Other scope of technologies (separated by commas)	1 (2.0%)	4 (8.0%)
**b) Depth of HTA use in pricing and/or reimbursement decision of health technologies**		
HTA is not applied to any health technologies	33 (60.8%)	6 (11.8%)
Only new technologies with significant budget impact	15 (29.4%)	9 (15.7%)
Only new technologies	2 (3.9%)	5 (9.8%)
New technologies + revision of previous pricing and reimbursement decisions	3 (5.9%)	34 (62.7%)
**5. Decision criteria**		
**a) Decision categories (multiple choice)**		
None of the below categories are applied	17 (33.3%)	3 (2.0%)
Unmet medical need	12 (19.6%)	33 (62.7%)
Health care priority	9 (15.7%)	40 (76.5%)
Assessment of therapeutic value	19 (35.3%)	40 (78.4%)
Cost-effectiveness	21 (39.2%)	39 (82.4%)
Budget impact	18 (33.3%)	42 (84.3%)
Other decision categories	0 (0.0%)	1 (2.0%)
**b) Decision thresholds**		
Thresholds are not applied	36 (70.0%)	3 (5.9%)
Implicit thresholds are preferred	11 (22.0%)	8 (15.7%)
Explicit soft thresholds are applied in decisions	4 (6.0%)	27 (51.0%)
Explicit hard thresholds are applied in decisions	1 (2.0%)	15 (27.5%)
**c) Multi-criteria decision analysis**		
No explicit multi criteria decision framework is applied	48 (98.0%)	8 (14.3%)
Explicit multi criteria decision framework is applied	1 (2.0%)	44 (85.7%)
**6. Quality and transparency of HTA implementation**		
**a) Quality elements of HTA implementation (multiple choice)**		
None of the below quality elements are applied	38 (77.6%)	4 (6.1%)
Published methodological guidelines for HTA/economic evaluation	4 (8.2%)	24 (53.1%)
Regular follow-up research on HTA recommendations	3 (6.1%)	23 (44.9%)
Checklist to conduct formal appraisal of HTA reports or submissions exists but not available for public	6 (10.2%)	19 (36.7%)
Published checklist is applied to conduct formal appraisal of HTA reports or submissions	0 (0.0%)	34 (67.3%)
**b) Transparency of HTA in policy decisions**		
Technology assessment reports, critical appraisal and HTA recommendation are not published	41 (81.6%)	3 (6.0%)
HTA recommendation is published without details of technology assessment reports and critical appraisal	6 (10.2%)	6 (12.0%)
Transparent technology assessment reports, critical appraisals and HTA recommendations	4 (8.2%)	44 (82.0%)
**c) Timeliness**		
HTA submission and issuing recommendation have no transparent timelines	42 (85.4%)	6 (12.0%)
HTA submissions are accepted/conducted following a transparent calendar, but issuing recommendation has no transparent timelines	6 (12.5%)	5 (10.0%)
HTA submissions are accepted continuously and issuing recommendation has transparent timelines	1 (2.1%)	42 (78.0%)
**7. Use of local data**		
**a) Requirement of using local data in technology assessment**		
No mandate to use local data	43 (84.0%)	4 (8.3%)
Mandate of using local data in certain categories without need for assessing the transferability of international evidence	4 (8.0%)	7 (14.6%)
Mandate of using local data in certain categories with need for assessing the transferability of international evidence	4 (8.0%)	39 (77.1%)
**b) Access and availability of local data**		
Limited availability or accessibility to local real-world data	43 (82.4%)	5 (9.8%)
Up-to-date patient registries are available in certain disease areas, but payers' databases are not accessible for HTA doers	7 (13.7%)	4 (7.8%)
Payers' databases are accessible for HTA doers, patient registries are not available or accessible in the majority of disease areas	2 (2.0%)	6 (11.8%)
Up-to-date patient registries are available in certain disease areas and payers' databases are accessible for HTA doers	1 (2.0%)	39 (70.6%)
**8. International collaboration**		
**a) International collaboration, joint work on HTA (joint assessment reports) and national/regional adaptation (reuse) (multiple choice)**		
No involvement into joint work; and no reuse of joint work or national/regional HTA documents from other countries	36 (75.0%)	2 (4.3%)
Active involvement in joint work (e.g., EUnetHTA Rapid REA, full Core HTA)	6 (8.3%)	20 (43.5%)
National/regional adaptation (reuse) of joint HTA documents	9 (18.8%)	27 (56.5%)
National/regional adaptation (reuse) of national/regional work performed by other HTA bodies in other countries	1 (2.1%)	36 (71.7%)
**b) International HTA courses for continuous education on HTA**		
Limited interest in (1) developing/implementing of and (2) participating at international HTA courses	31 (60.0%)	6 (11.8%)
Interest only in regular participation at international HTA courses	12 (22.0%)	2 (3.9%)
High interest in (1) developing/implementing of and (2) participating at international HTA courses	9 (18.0%)	46 (84.3%)

### Capacity Building

As HTA implementation necessitates highly skilled professionals in a multidisciplinary field and capacity building of human resources is a critical element of HTA roadmaps.

Limited current options for HTA training are indicated by 65% of respondents. Indeed, project-based HTA workshops or short courses—usually sponsored by pharmaceutical companies—are still the most common form of HTA education in the MENA region, which may not be sufficient to induce hands-on training experience. On the other hand, capacity building should not focus only on “HTA doers” with advanced technical skills, because without decision makers' understanding and commitment HTA implementation cannot be accelerated. As a good example, INEAS, the Tunisian HTA body invested in both training of its own staff and eminent representatives of different stakeholder groups, including policy makers and clinicians. Training of potential “HTA users” improves general understanding on what can and what should not be expected from HTA implementation, how to frame and scope HTA in countries with limited HTA resources and increase the impact of HTA deliverables in decision-making.

Undergraduate courses are not optimal for in-depth HTA training, especially for the methodology of economic modeling. However, they are important options to raise the awareness, positive attitude, basic knowledge, and understanding on the potential contribution of health care professionals to the HTA process. Medical and pharmaceutical education is strong and well established in the MENA region. Furthermore, many health care managers in the region hold MBA degrees, still, these courses have limited HTA related components currently. Some universities in Egypt, Jordan, or Lebanon have already launched HTA related subjects to the curriculum of pharmacy or economic undergraduate training.

Another way to build capacity is holding intensive training programs within a specific organization, such as the HTA Department of the Ministry of Health or even a large-scale hospital. The capacity building program for hospital pharmacists at King Hussein Cancer Center in Jordan was established based on needs assessment to tackle the scarcity of HTA analysts (Al Rabayah et al., 2018). The Jordanian experience shows that a structured HTA capacity‐building program can be developed and implemented even in countries with limited resources. In the future, 76% of survey respondents would prefer having permanent postgraduate HTA training (supported by other training tools) in their own countries.

Sending students to acquire postgraduate training abroad (e.g., in Europe or North America) is a common practice in several MENA countries to induce the capacity building process. However, it is not a sustainable long-term solution to overcome limited human resources due to the high cost of living and training abroad and because talented postgraduates may pursue career opportunities in more developed countries, and never return to their own region. This approach may be reasonable to train those future trainers, who can help to establish and lead local academic programs (Kaló et al., 2013).

If HTA is mandated in local pharmaceutical pricing and reimbursement decisions with a bottleneck on local training opportunities, experienced HTA professionals from the public sector may move to the pharmaceutical industry. The brain drain can be reduced by increased output from local postgraduate courses, which are accessible at fairly low cost for young talents.

Such postgraduate HTA trainings have recently started in Egypt, where the first postgraduate diploma in health economics with core HTA components was established in 2012 at the Graduate School of the Arab Academy of Science and Technology, followed in 2014 by a master program provided in collaboration between the Public Health Department at Faculty of Medicine and Faculty of Political Science and Economics at Cairo University. In 2017, the Faculty of Pharmacy at Monastir in Tunisia launched a post-graduate program in pharmacoeconomics, market access, and HTA by inviting lecturers from INEAS and the international HTA community. A Market Access of Health Products master program at the Lebanese University in Beirut was launched in 2014 with courses on Pharmacoeconomics, Pricing and reimbursement of pharmaceuticals, and Introduction to HTA.

### Funding HTA

The success of HTA implementation partially depends on how much financial resources are invested into both HTA phases. The assessment phase focuses on rigorous review and synthesis of scientific evidence, which is followed by the appraisal phase, the contextualization of assessment results (Kristensen et al., 2019).

Highly limited funding for HTA assessment was indicated by 52% of respondents while private funding was reported mainly by 38%. Pharmaceutical manufacturers may have an interest in referring to HTA evidence from those countries, where market access of their products is already granted. Some companies have already invested in HTA research in the region, hence, limited sporadic private funding is available in selected MENA countries.

In the future, the majority of respondents prefer less dependence on private investment to HTA, hence 39% expect sufficient and another 39% dominant public funding for HTA research. Reasonable public investment into HTA research is desirable even in less affluent MENA countries, as public funding does not only ensure the sustainability of HTA and reduce conflict of interest but also indicates the political will and the conviction of governmental officials to HTA implementation and its use for decision making in health care. In addition, public funding is necessary to review the appropriateness of previous HTA decisions or conduct multiple technology assessments in which several technologies of different manufacturers are evaluated in priority disease areas.

On the other hand, several middle-income countries, including those in Central and Eastern Europe decided that private industry should be responsible for generating and funding HTA evidence in submission dossiers, and public bodies should mainly be responsible for critically appraising the submitted evidence. In countries with severe resource constraints, the Scottish Medical Consortium (SMC) is often referenced in policy discussions, as the HTA organization in Scotland is quite efficient compared to its fairly small size and low budget (Kaló et al., 2016).

Highly limited funding for the appraisal process was reported by 78% of respondents. In those countries, where there is no dedicated agency to review HTA evidence submitted by pharmaceutical manufacturers, critical appraisal is expected by non-paid HTA committee members. If an HTA agency is established with highly trained employees, more budget is needed for the critical appraisal process. In the future, 22% of survey respondents expect private funding (e.g., submission fees by pharmaceutical manufacturers), and 71% expect public funding (e.g., budget from general government revenues) for the critical appraisal.

### Legislation on HTA

Capacity building and securing funding are essential for HTA implementation, but without embedding HTA in the legislation, in other words, making HTA an obligatory step for either pricing or reimbursement of new technologies, the efforts would go in vain. 55% of respondents indicated that HTA did not have a formal role in their countries, while 36% reported that mainly international HTA evidence was considered in policy decisions. Indeed, some experts believe that in resource-constrained countries policymakers may improve evidence of their decisions by relying on HTA recommendations from other countries (Lopert et al., 2013; Dankó, 2014). Still, 87% of respondents disagreed with this approach by highlighting the need for local HTA evidence in the future.

If the HTA process considers local evidence, a committee may not be sufficient for the appraisal of HTA dossiers. 59% of respondents reported that HTA was not institutionalized in their countries, while 24% reported that only an HTA committee was responsible for providing HTA input into policy decisions in their countries.

In the future, 80% of respondents prefer either a single HTA agency or multiple agencies. The centralization or decentralization of the HTA body is highly dependent on the country size, fragmentation of health care financing, HTA capacities, and readiness of health care systems. Academic support of HTA agencies is more appropriate in those countries, where postgraduate HTA training is available.

Institutionalization of HTA has already been initiated in selected MENA countries. In Saudi Arabia, mandatory HTA for high-cost drugs was initiated through the High-Cost Medication Committee under Saudi Health Council, supported by a forthcoming HTA center under the Ministry of Health, which has already been budgeted with ongoing implementation. HTA programs have also been initiated at the Drug Policy & Economics Center under Ministry of National Guard Health Affairs as part of the 2020 National Transformation Program. In Egypt, a pharmacoeconomics unit was officially established in 2011 under the Central Administration of Pharmaceutical Affairs within the Ministry of Health, but HTA is not obligatory for pricing or reimbursement, However, the recent universal health insurance law from 2018 mandates the representation of health economists in the governing boards of the new Universal Health Insurance Authority and the health care provider body. In Tunisia, the central HTA body (INEAS) represents a national authority under the auspices of the Ministry of Health with the objective of assessing the added benefit and cost-effectiveness of health technologies and providing rigorous evidence-based recommendations to decision-makers on pharmaceuticals and other technologies' uptake and use. In 2016, the Lebanese Ministry of Public Health (MoPH) drew a Health Strategic Plan for the medium term with a focus on Health Technology Assessment systems and procedures. Jordan started hospital level HTA at King Hussein Cancer Center (KHCC). The Center for Drug Policy and Technology Assessment at KHCC is responsible for conducting HTAs, their assessments are appraised by the Pharmacy and Therapeutic (P&T) Committee and HTA results are currently utilized to support formulary listing decisions.

### Scope of HTA

HTA was not applied to any health technologies in their countries as stated by 51% of respondents, 49% reported that HTA was utilized to support decisions related to pharmaceuticals, and few reported the current use of HTA for other health technologies. In the future, the majority of respondents prefer extending the scope of HTA to different technologies, including pharmaceuticals (92%), medical devices (78%) prevention programs (66%), and surgical interventions (64%). Respondents with percentage of 63% believe that, in the long-run, HTA should not be restricted only to new interventions, but should be extended to the revision of previous HTA recommendations because once a technology goes into the benefit package, it is hard to remove it without HTA evidence.

HTA capacity at the early phases may not be sufficient to support a wide range of services or decision domain, so prioritization is needed. It is important to start embedding HTA in the legislation, even if it does not cover a wide scope of services or decisions. First HTA can be used to advice new pharmaceuticals with high expected budget impact, similar to what has been done in some MENA countries. In Tunisia, INEAS currently focuses on technologies with high cost or an important impact on the Tunisian Health System. At King Hussein Cancer Center (KHCC) in Jordan, HTA is targeted to expensive pharmaceuticals. Also, Saudi Arabia has started to use HTA evidence at the national High-Cost Drugs Committee. Once sufficient HTA capacities are available and different stakeholders are aligned about the HTA process, the scope of HTA can be extended to cover all new pharmaceuticals, partly because it is easier to synthesize clinical evidence for pharmaceutical therapies due to the mandatory registration trials. In the next phase, HTA evidence can be mandated for medical devices and other health technologies, and revision of previous HTA recommendations can also be considered.

WHO is playing an important role in framing and scoping of HTA. The Regional Office for the Eastern Mediterranean has initiated technical missions in several MENA countries to assist Ministries of Health in improving the process for evidence-based health policy decision making.

### Decision Criteria

Health technology assessment has multiple domains, and individual countries may not necessarily consider all domains in their policy process. As indicated by 33% of respondents, their own country did not consider any decision categories. The most common decision criteria are cost-effectiveness (39%), therapeutic value (35%), and budget impact (33%). In the future, participants prefer considering more categories for decision making including health care priority (76%), therapeutic value (78%), cost-effectiveness (82%), budget impact (84%), and unmet medical need (63%).

Justification of policy decisions based on HTA results can be improved, if decision rule in selected domains of HTA is determined. Decision thresholds are generally applied for the cost-effectiveness criterion. The major differences across countries are 1) whether the threshold is published (i.e., explicit) or not, 2) whether the hard threshold is used as a rule (e.g., if a technology is not cost-effective, it cannot be reimbursed) or the soft threshold is applied as a tool to negotiate about price reductions (e.g., not cost-effective technologies still can be reimbursed with managed entry agreements), and 3) how the threshold is established. Incremental cost-effectiveness ratios may be compared to the economic status of countries, which was initially applied in the WHO-CHOICE project (Hutubessy et al., 2003). Although WHO does not recommend this practice anymore (Garner et al., 2018), GDP per capita is still the most frequently applied cost-effectiveness threshold (Cameron et al., 2018). Respondents with percentage of 70% reported no thresholds in their countries. In the future, the vast majority of respondents would like to have some sort of a threshold, 51% of them prefer selecting an explicit soft threshold.

Multicriteria decision analysis (MCDA) is increasingly used in health care globally to improve the consistency and transparency of policy decisions (Thokala et al., 2016). Although MCDA has been applied only in pilot studies in the MENA region (Fouad and ElMordy, 2017; Abdullah et al., 2019), in the future, the majority of respondents (86%) consider the broader utilization of this methodology.

### Quality and Transparency

Quality of HTA can be improved by multiple approaches. There are 78% of respondents who were not aware of using any tools for quality improvement in their countries. In the future, however, respondents would prefer having published methodological guidelines (53%), follow-up on HTA recommendations (45%), internal checklist for the critical appraisal of submitted HTA reports (37%), or even published critical appraisal checklist to allow HTA doers to conduct self-appraisal of their dossiers before submission (67%).

Leaders of ISPOR Egypt Chapter and the Pharmacoeconomics Unit of the Ministry of Health published recommendations for reporting pharmacoeconomic evaluations in Egypt (Elsisi et al., 2013). In Tunisia, INEAS published HTA submission guidance (clinical part) for pharmaceutical companies (INEAS, 2019). In Lebanon the Ministry of Health, the National Social Security Funds in collaboration with the Lebanese University developed the first Lebanese pharmacoeconomics guidelines. In Jordan, King Hussein Cancer Center has developed a formulary submission pathway that considers both the clinical and pharmacoeconomic aspects.

Transparency of HTA documents is an integral component of justifiable policy decisions. As indicated by 82% of respondents, these documents were not in the public domain in their countries, however, they prefer publication of technology assessment reports critical appraisals and HTA recommendations. Tunisia is a good example of HTA transparency, as HTA projects and reports are published on the INEAS website.

Timeliness of HTA is a key element to improve the predictability of evidence-based policy decisions. As indicated by 85% of respondents, limited transparency of HTA timelines, however, in the future 78% of respondents advocate that HTA submissions should be accepted continuously and issuing recommendation should have transparent timelines.

### Local Data

Transferring good quality international evidence—typically about the relative effectiveness of technologies—could be beneficial and save resources for local HTAs (Kleijnen et al., 2014). However, making decisions based on international HTA recommendations without considering limitations of transferability (especially related to treatment costs) makes more harm than good. Certain elements of HTA reports are transferable, but the adjustment to local data is necessary (Kaló et al., 2012).

As reported by 84% of respondents, the use of local data in the HTA process was not mandated in their countries, while in the future the majority (77%) preferred the mandate of using local data in certain categories with the need for assessing the transferability of international evidence.

Survey results highlighted the limitations of local data for conducting HTA in MENA countries due to limited availability of patient registries and restricted access to payers' databases. Although the deficiency of high-quality local data is another barrier in the region currently, efforts to collect and use local data may teach HTA doers on how to improve its quality, especially in countries with significant investment into the information technology infrastructure of health care financing and provision. In the future, 82% of respondents would invest in patient registries and make payers' databases available for HTA doers.

### International Collaboration

Duplication of efforts in HTA research should be avoided, hence international collaboration among HTA bodies can be highly beneficial. As opposed to the current situation where the majority of the respondents (75%) reported no involvement into joint international work, almost all (96%) opted for some sort of international collaboration either by active involvement in joint work initiatives or reuse of HTA materials prepared by distinguished international HTA bodies. Efforts of the European Union to facilitate HTA collaboration provides useful experience on how to develop methodology and network for joint HTA work (Kristensen et al., 2009). On the other hand, joint HTA assessment can also be done for specific technologies in developing countries with more modest investment (Pichon-Riviere et al., 2015).

International collaboration may also contribute to the process of capacity building, hence 84% of respondents would prefer developing and/or participating at international HTA courses. In Tunisia, INEAS joined the International Network of Agencies in Health Technology Assessment (INAHTA) in 2015 to facilitate international collaboration.

## Discussion

Currently, HTA implementation is still in the early stage with some heterogeneity in MENA countries. The preferred status seems to be more homogenous, yet we have to take this with a grain of salt, although the destination is similar, the route can be different from one country to another. Therefore, each MENA country should develop its context-specific HTA roadmap, as such roadmaps are not transferable without considering country size, GDP per capita, major social values, public health priorities and adopted systems of health care financing (Hamidi and Akinci, 2016). These roadmaps should include long-term objectives in all major areas of HTA implementation, and an action plan with clear timelines. It is not sufficient only to design the roadmap, constant monitoring of the HTA implementation is recommended, which may necessitate the readjustment of timelines or even action items.

Our results should be viewed as an initial step in a multi-stakeholder dialogue on HTA implementation in the MENA region. The generalizability of our main conclusions is limited, and they should be validated in further policy research studies, partly because we had no opportunity to ensure full and proportional representation of each country and all stakeholder groups in the MENA region among survey participants and the discussion group. Similarly, as our focus was on regional commonalities, further policy research should explore differences across MENA countries in their HTA implementation roadmaps. In fact, the sample size in our survey was relatively small, which prevented us from comparing answers of different type of respondents or different countries. Yet, responses were homogenous especially when it came to the preferred status in 10 years. Hence, we can conclude that health policy experts in MENA countries would like to facilitate HTA implementation and expect significant changes in 10 years compared to the current status. The most important generalizable conclusions — based on the survey results and validated by coauthors — are presented in **Table 3**.

**Table 3:** Summary on generalizable conclusions about HTA implementation in the MENA region.

HTA Capacity Building	More graduate and postgraduate HTA trainings have to be developed on the basis of country-specific needs.
HTA Funding	Increased public budget is needed for HTA research and the critical appraisal of HTA submissions
Legislation on HTA	There are two main options for the institutionalization of HTA: • a central HTA agency with the support of academic networks • establishment of multiple HTA bodies within a country preferably with central coordination
Scope of HTA Implementation	The scope of HTA has to be extended • from pharmaceuticals to non-pharmaceutical technologies • to revision of previous policy decisions
Decision criteria	Although cost-effectiveness with explicit threshold remains the most preferred HTA criterion, several other criteria have to be considered, maybe even by applying an explicit MCDA framework.
Quality and transparency of HTA implementation	The quality of HTA work have to be improved by applying multiple methods. Publication of HTA deliverables and timeliness of HTA processes have to be ensured.
Use of local data	In policy decisions the role of local evidence and data has to be strengthened, which translates to the extended use of local patient registries and payers' databases.
International collaboration	Duplication of efforts can be reduced if international collaboration is integrated into national HTA implementation.

The local political will may be the most important driver for changing, so health policy experts and HTA professionals should be able to explain the rationale and potential benefits of HTA investment to political leaders.

Although HTA systems and implementation plans are not fully transferable due to several factors, relevant international practices still should be considered in designing country-specific roadmaps. The ISPOR HTA Council Working Group Report highlighted that many good practices had been developed in areas of assessment and some other key aspects of defining HTA processes (Kristensen et al., 2019). The World Health Organization Regional Office for the Eastern Mediterranean (WHO EMRO) can facilitate this process by providing unbiased and politically neutral guidance into national health policies. On the other hand, regional initiatives, such as the Middle East and North Africa Health Policy Forum *(MENA HPF)* may also support HTA implementation by serving as a think tank, and platform for networking and advocacy for health policy and decision makers in the region. Another potential regional collaboration in HTA can be through the Health Council of the Gulf Cooperation Council (About Gulf Joint Procurement Program, 2018) which facilitates key stakeholders from different countries to share best practices or even negative experiences.

## Data Availability Statement

All datasets generated for this study are included in the article/**Supplementary Material**.

## Author Contributions

All authors contributed to this work and approve the version to be published and are also accountable for all aspects of the work.

## Conflict of Interest

The authors declare that the research was conducted in the absence of any commercial or financial relationships that could be construed as a potential conflict of interest.
