# Supplementary material for: Implementation of Health Technology Assessment in the Middle East and North Africa: Comparison Between the Current and Preferred Status
Source: Front Pharmacol. 2020 Feb 21;11:15. doi: 10.3389/fphar.2020.00015 (PMC7046555; doi:10.3389/fphar.2020.00015)
Supplement: Supplementary file 1 [file DataSheet_1.docx]

**Appendix: HTA implementation scorecard**

**Comparison of current status and future directions**

A scorecard was designed to assess the current status of HTA implementation in the region of Middle East and North Africa (MENA) and to set up long-term objectives. Our scorecard must be viewed as an initial step in a multi-stakeholder dialogue on identifying best practices, common problems and proposing further directions in the MENA region.

Please provide answers by checking boxes or radio buttons for your own country. For “single choice” questions please select only 1 option for current status and 1 option for preferred status in 10 years. Please provide answer for all questions. For “multiple choice” questions please select all relevant options related to your country, or the first option when none of the listed options are relevant to your country. In most cases you may also select “other” to indicate additional options.

The anonymity of respondents is guaranteed. Aggregated results will be presented first at the ISPOR Dubai 2018 conference, then in a peer-reviewed scientific journal.

Should you need further information about the terminology in the survey, please check the below paper:

*Kaló Z, Gheorghe A, Huic M, Csanádi M, Kristensen FB. HTA implementation roadmap in Central and Eastern European countries. Health Econ. 2016. 25. S1. 179–192. (available at* [*https://doi.org/10.1002/hec.3298*](https://doi.org/10.1002/hec.3298)*)*

| **Consent** | **yes/no** |
| --- | --- |
| Hereby, I accept that my anonym answers can be aggregated and presented in educational events. |  |
| Hereby, I accept that my anonym answers can be aggregated and used in scientific presentations and publications. |  |

**HTA implementation scorecard**

**Please provide answers about your own background**

***(individual survey results are kept strictly confidential)***

| **Country** |  |  |
| --- | --- | --- |
| **Please indicate your country: ………………………………………………………………………..** |  |  |
|  |  |  |
| **Main employment** |  |  |
| **Public sector** |  |  |
| - Decision-maker, policymaker, public payer, Ministry of Health (potential HTA user) |  |  |
| - HTA agency |  |  |
| - Academic sector |  |  |
| - Public health care provider (e.g. clinician) |  |  |
| - Other (please explain): ………………………………………… |  |  |
| **Private sector** |  |  |
| - Health care industry (e.g. pharmaceutical or medical device company) |  |  |
| - Private health care provider (e.g. clinician) |  |  |
| - Pharmaceutical trade sector (e.g. wholesaler, pharmacy) |  |  |
| - Private insurance - Consultancy |  |  |
| - Journalist |  |  |
| - Other (please explain): …………………………………………. |  |  |
|  |  |  |
| **Major training (single choice)** |  |  |
| - Economics |  |  |
| - Pharmacy |  |  |
| - Medicine |  |  |
| - Other health care (e.g. nursing, dietetics) |  |  |
| - Multidisciplinary (at least two master’s degrees from the above list) |  |  |
| - Other (please explain): …………………………………………. |  |  |
|  |  |  |
| **Age** |  |  |
| - Below 30 |  |  |
| - Between 30-50 |  |  |
| - Above 50 |  |  |

|  |  |  |
| --- | --- | --- |
| **1. HTA capacity building**  **Education (*single choice*)** | **current status** | **preferred status in 10 years** |
| - No training |  |  |
| - Project based training and short courses |  |  |
| - Permanent graduate program with short courses |  |  |
| - Permanent graduate and postgraduate program with short courses |  |  |
|  |  |  |
| **2. HTA funding** |  |  |
| **Financing critical appraisal of technology assessment (*single choice*)** |  |  |
| - No funding for critical appraisal of technology assessment reports or submissions |  |  |
| - Dominantly private funding (e.g. submission fees) by manufacturers for the critical appraisal of technology assessment reports or submissions |  |  |
| - Dominantly public funding for critical appraisal of technology assessment reports or submissions |  |  |
| **Financing health technology assessment (i.e. HTA research) (*single choice*)** |  |  |
| - No public funding for technology assessment; private funding is not needed or expected |  |  |
| - No or marginal public funding for research in HTA; private funding is expected |  |  |
| - Sufficient public funding for research in HTA; private funding is also expected |  |  |
| - HTA research is dominantly funded from public resources |  |  |
|  |  |  |
| **3. Legislation on HTA** |  |  |
| **Legislation on the role of HTA process and recommendations in decision-making**  **process (*single choice*)** | | |
| - No formal role of HTA in decision-making |  |  |
| - Dominantly international HTA evidence is taken into account in decision-making |  |  |
| - International and additionally local HTA evidence is taken into account in decision-making |  |  |
| - Local HTA evidence is mandatory in decision making |  |  |
| **Legislation on organizational structure for HTA appraisal (*single choice*)** |  |  |
| - There is no public committee or institute for the appraisal process |  |  |
| - Committee is appointed for the appraisal process |  |  |
| - Committee is appointed for the appraisal process with support of academic centers and independent expert groups |  |  |
| - A public HTA institute or agency is established to conduct formal appraisal of HTA reports or submissions |  |  |
| - Public HTA institute or agency is established to conduct formal appraisal of HTA reports or submissions with support of academic centers and independent expert groups |  |  |
| - Several public HTA bodies are established without central coordination of their activities |  |  |
| - Several public HTA bodies are established with central coordination of their activities |  |  |
|  |  |  |
| **4. Scope of HTA implementation** | **current status** | **preferred status in 10 years** |
| **Scope of technologies (*multiple choice*)** |  |  |
| - HTA is not applied to any health technologies |  |  |
| - Pharmaceutical products |  |  |
| - Medical devices |  |  |
| - Prevention programs and technologies |  |  |
| - Surgical interventions |  |  |
| - Other (please specify): ...................................... |  |  |
| **Depth of HTA use in pricing and/or reimbursement decision of health technologies (*single choice*)** | | |
| - HTA is not applied to any health technologies |  |  |
| - Only new technologies with significant budget impact |  |  |
| - Only new technologies |  |  |
| - New technologies + revision of previous pricing and reimbursement decisions |  |  |
|  |  |  |
| **5. Decision criteria** |  |  |
| **Decision categories (*multiple choice*)** |  |  |
| - None of the below categories are applied |  |  |
| - Unmet medical need |  |  |
| - Health care priority |  |  |
| - Assessment of therapeutic value |  |  |
| - Cost-effectiveness |  |  |
| - Budget impact |  |  |
| - Other (please specify): ……………………………………. |  |  |
| **Decision thresholds (*single choice*)** |  |  |
| - Thresholds are not applied |  |  |
| - Implicit thresholds are preferred |  |  |
| - Explicit soft thresholds are applied in decisions |  |  |
| - Explicit hard thresholds are applied in decisions |  |  |
| **Multi criteria decision analysis (*single choice*)** |  |  |
| - Explicit multi criteria decision framework is applied |  |  |

| **6. Quality and transparency of HTA implementation**  **Quality elements of HTA implementation (*multiple choice*)** |  |  |
| --- | --- | --- |
| - None of the below quality elements are applied |  |  |
| - Published methodological guidelines for HTA/economic evaluation |  |  |
| - Regular follow-up research on HTA recommendations |  |  |
| - Checklist to conduct formal appraisal of HTA reports or submissions exists but **not available** for public |  |  |
| - Published checklist is applied to conduct formal appraisal of HTA reports or submissions |  |  |

| **Transparency of HTA in policy decisions (*single choice*)** | **current status** | **preferred status in 10 years** |
| --- | --- | --- |
| - Technology assessment reports, critical appraisal and HTA recommendation are **not published** |  |  |
| - HTA recommendation is published without details of technology assessment reports and critical appraisal |  |  |
| - Transparent technology assessment reports, critical appraisals and HTA recommendations |  |  |
| **Timeliness (*single choice*)** |  |  |
| - HTA submission and issuing recommendation have no transparent timelines |  |  |
| - HTA submissions are accepted/conducted following a transparent calendar, but issuing recommendation has no transparent timelines |  |  |
| - HTA submissions are accepted continuously and issuing recommendation has transparent timelines |  |  |
|  |  |  |
| **7. Use of local data** |  |  |
| **Requirement of using local data in technology assessment (*single choice*)** |  |  |
| - No mandate to use local data |  |  |
| - Mandate of using local data in certain categories **without** need for assessing the transferability of international evidence |  |  |
| - Mandate of using local data in certain categories **with** need for assessing the transferability of international evidence |  |  |
| **Access and availability of local data (*single choice*)** |  |  |
| - Limited availability or accessibility to local real world data |  |  |
| - Up-to-date patient registries are available in certain disease areas, but payers’ databases are not accessible for HTA doers |  |  |
| - Payers’ databases are accessible for HTA doers, patient registries are not available or accessible in the majority of disease areas |  |  |
| - Up-to-date patient registries are available in certain disease areas and payers’ databases are accessible for HTA doers |  |  |
| **8. International collaboration** |  |  |
| **International collaboration, joint work on HTA (joint assessment reports) and national/regional adaptation (reuse) *(multiple choice)*** |  |  |
| - No involvement into joint work; and no reuse of joint work or national/regional HTA documents from other countries |  |  |
| - Active involvement in joint work (e.g. EUnetHTA Rapid REA, full Core HTA) |  |  |
| - National/regional adaptation (reuse) of joint HTA documents |  |  |
| - National/regional adaptation (reuse) of national/regional work performed by other HTA bodies in other countries |  |  |
| **International HTA courses for continuous education on HTA *(single choice)*** |  |  |
| - Limited interest in (1) developing / implementing of and (2) participating at international HTA courses |  |  |
| - Interest only in regular participation at international HTA courses |  |  |
| - High interest in (1) developing / implementing of and (2) participating at international HTA courses |  |  |
